# From prediction to actionable mechanisms: Explainable multi‑omics AI for farm‑to‑fork postharvest preservation

**DOI:** 10.1002/imt2.70139

**Published:** 2026-06-09

**Authors:** Peihua Ma, Xiaoxue Jia, Bei Fan, Boqiang Li, Tao Lin, Jiping Sheng, Cheng‐I Wei, Yingjian Lu, Yizhou Ma, Lin Chen, Songtao Jiu, Fengzhong Wang

**Affiliations:** ^1^ Institute of Food Science and Technology, Chinese Academy of Agricultural Sciences, Key Laboratory of Agro‐products Processing Ministry of Agriculture and Rural Affairs Beijing China; ^2^ State Key Laboratory of Plant Diversity and Specialty Crops, Institute of Botany Chinese Academy of Sciences Beijing China; ^3^ Beijing Key Laboratory of Growth and Developmental Regulation for Protected Vegetable Crops, College of Horticulture China Agricultural University Beijing China; ^4^ School of Agricultural Economics and Rural Development Renmin University of China Beijing China; ^5^ Department of Nutrition and Food Science, College of Agriculture and Natural Resources University of Maryland, College Park College Park Maryland USA; ^6^ College of Food Science and Engineering Nanjing University of Finance and Economics Nanjing China; ^7^ Laboratory of Food Process Engineering Wageningen University & Research Wageningen The Netherlands; ^8^ School of Chemistry, Chemical Engineering and Biotechnology Nanyang Technological University Singapore Singapore; ^9^ Department of Plant Science, School of Agriculture and Biology Shanghai Jiao Tong University Shanghai China

## Abstract

Graphical overview of explainable artificial intelligence (XAI) for farm‐to‐fork postharvest preservation. Postharvest deterioration accumulates across orchard, packhouse, refrigerated transportation, warehouse, and distribution stages under fluctuating temperature, humidity, atmosphere, and mechanical stress. Multimodal data streams, including host omics, microbiome profiles, environmental sensing, RGB/hyperspectral/thermal imaging, spectroscopy, key genes, and logistics records, are integrated through a data lakehouse and analyzed by postharvest XAI models. Explainable modules, including SHapley Additive exPlanations (SHAP)/local attribution, graph neural network (GNN) explanation, pathway‐constrained models, counterfactual reasoning, and stability/faithfulness auditing, convert black‐box spoilage‐risk prediction into interpretable biological mechanisms. These mechanisms guide actionable interventions such as antioxidant coating, elicitor spray, biocontrol consortia, packaging optimization, and gene‐targeted strategies. Validation through storage trials, sensory evaluation, microbial testing, and sequencing closes the loop from prediction to explanation, intervention, and validated decision support.

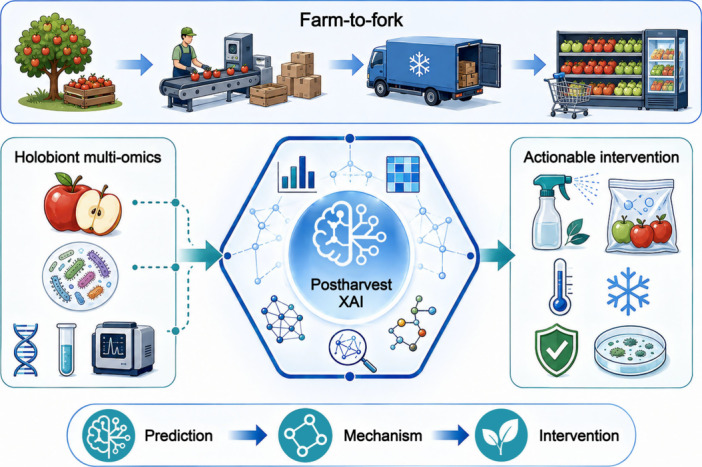

## AUTHOR CONTRIBUTIONS

**Peihua Ma**: Conceptualization; methodology; data curation; writing—original draft; writing—review and editing; visualization. **Xiaoxue Jia**: Writing—original draft; writing—review and editing. **Bei Fan**: Writing—review and editing; supervision. **Boqiang Li**: Writing—review and editing. **Tao Lin**: Writing—review and editing. **Jiping Sheng**: Writing—review and editing. **Cheng‐I Wei**: Writing—review and editing. **Yingjian Lu**: Writing—review and editing. **Yizhou Ma**: Writing—review and editing; data curation; validation. **Lin Chen**: Writing—review and editing. **Songtao Jiu**: Writing—review and editing. **Fengzhong Wang**: Writing—review and editing; project administration; supervision; resources; funding acquisition.

## CONFLICT OF INTEREST STATEMENT

The authors declare no conflicts of interest.

## ETHICS STATEMENT

No animals or humans were involved in this study.

To the Editor,

Postharvest deterioration remains a critical inefficiency in global food systems, with 13.3% of food lost between harvest and retail, losses exceeding 25% for fruits and vegetables, and an additional 19% wasted at the consumer level. These losses exacerbate greenhouse gas emissions, resource depletion, and food insecurity, reinforcing the urgency of advancing preservation strategies [1, 2, 3]. By integrating phenotypic monitoring with genomic, transcriptomic, and microbiome data, AI has progressed from visual sorting to molecular‐level quality assessment, improving the precision of spoilage prediction and shelf‐life estimation [4]. However, most current AI models deliver forecast predictions without elucidating the underlying biological or environmental mechanisms. This lack of interpretability limits actionable decision‐making, as effective intervention depends on understanding the drivers of deterioration rather than prediction alone.

Explainable artificial intelligence (XAI) addresses this limitation by making model reasoning interpretable and translating complex outputs into actionable knowledge [5, 6]. XAI frameworks are commonly categorized as post‐hoc versus intrinsic methods: post‐hoc methods interrogate trained models to identify influential genes, metabolites, or storage variables, whereas intrinsic methods embed interpretability directly into model architecture, such as pathway‐constrained neural networks, reflect known regulatory networks governing ripening and spoilage. Importantly, applications in genomics and multi‐omics research demonstrate that XAI can reveal key genes, microbial taxa, and metabolic pathways driving predictions without sacrificing performance, thereby converting statistical associations into biologically meaningful hypotheses. In postharvest contexts, an explainable multi‐omics model could identify specific gene expression signatures or microbial components of the produce holobiont, defined as the host and its associated microbiota considered as an integrated biological unit, that are associated with accelerated spoilage and may guide targeted breeding, microbiome modulation, or environmental control strategies. Moreover, when integrating multimodal data, such as imaging, spectroscopy, and omics, XAI further disentangles the contribution of each modality, while counterfactual explanations and digital twin simulations allow evaluation of how modifying storage conditions might alter outcomes. Through these advances, explainability shifts AI from a predictive black box to a tool for mechanistic insight and actionable intervention [7].

**Figure 1:**
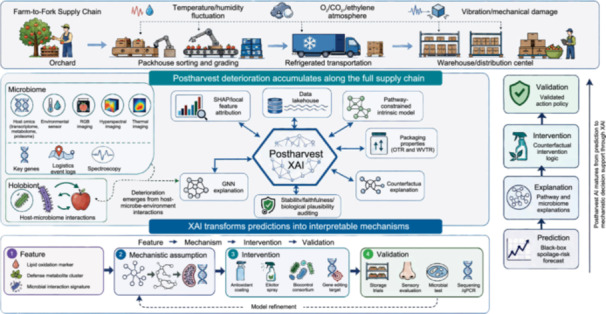
Concept of explainable AI in postharvest preservation within an XAI‐ready data ecosystem. Postharvest deterioration accumulates across orchard, packhouse, cold‐chain transport, storage, and distribution under fluctuating temperature, humidity, atmosphere, and handling stress. Multimodal inputs such as environmental sensors, RGB/hyperspectral/thermal imaging, spectroscopy, logistics events, host omics, microbiome profiles, and packaging properties are integrated in a data lakehouse. SHAP/local attribution, GNN explanation, pathway constraints, counterfactual reasoning, and stability–faithfulness audits convert spoilage prediction into interpretable mechanisms, guiding interventions and validation through storage trials, sensory assessment, qPCR, and sequencing. GNN, graph neural network; OTR, oxygen transmission rate; qPCR, quantitative polymerase chain reaction; RGB, red, green, blue; SHAP, SHapley Additive exPlanations; WVTR, water vapor transmission rate; XAI, explainable artificial intelligence.

This work, for the first time, synthesizes emerging advances to reposition postharvest AI from prediction‐centric analytics toward causality‐driven mechanism discovery (Figure 1). We introduce an XAI‐ready conceptual framework that integrates holobiont multi‐omics, multimodal sensing, and a data lakehouse architecture as a unified foundation for postharvest preservation research. This perspective establishes XAI as a critical scientific instrument for making postharvest AI not only mechanistically interpretable but also operationally actionable across the farm‐to‐fork continuum.

## FROM FOUNDATIONAL XAI CONCEPTS TO PROFESSIONAL DEPLOYMENT IN POSTHARVEST MULTI‐OMICS SYSTEMS

In postharvest fruit and vegetable preservation, XAI must progress from conceptual understanding to operational rigor, ultimately serving biological interpretation, mechanism discovery, and intervention design rather than merely enhancing model transparency. Below, we outline this progression across beginner, intermediate, and professional levels, explicitly framed within postharvest and multi‐omics research [8, 9, 10, 11] (See the detailed tutorial in Supporting Information). At the professional stage, XAI in postharvest research evolves from descriptive attribution toward intervention‐oriented decision frameworks that integrate counterfactual reasoning, robustness auditing, and biological validation [12, 13, 14, 15, 16].

## XAI‐READY DATA ECOSYSTEM FOR A POSTHARVEST APPLICATION

### Holobiont data ecosystem

**Figure 2:**
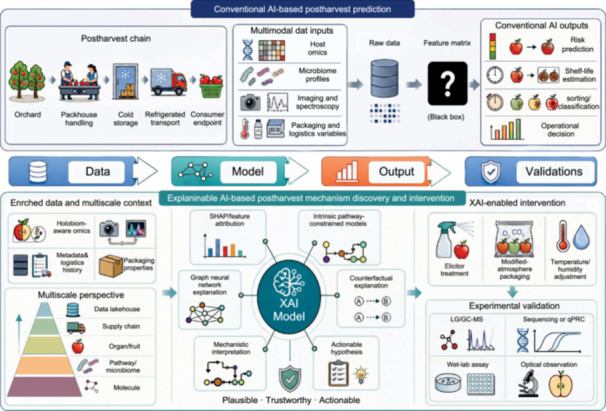
Comparison between conventional AI‐based postharvest prediction and XAI‐based mechanism discovery and intervention. Conventional AI integrates multimodal inputs from the postharvest chain but often produces black‐box outputs from feature matrices. In contrast, XAI enriches raw data with holobiont omics, logistics metadata, packaging properties, and multiscale context from molecules to supply chains. SHAP attribution, GNN explanation, pathway‐constrained modeling, counterfactual reasoning, and mechanistic interpretation generate actionable hypotheses, guiding elicitor treatment and experimental validation by LC/GC‐MS, wet‐lab assays, qPCR, and storage trials. LC/GC‐MS, liquid chromatography/gas chromatography‐mass spectrometry; qPCR, quantitative polymerase chain reaction; SHAP, SHapley Additive exPlanations.

Postharvest deterioration emerges from dynamic host‐microbe‐environment interactions rather than isolated fruit properties, driven by microbial succession and metabolic exchanges (Figure 2). This holobiont perspective dictates that an XAI‐ready data ecosystem must synchronize host omics, microbiome profiles, and environmental exposomes to capture these coupled dynamics, rather than storing them as siloed variables. Crucially, host transcriptional responses, metabolite accumulation, and microbiome succession often occur on different temporal scales and across different tissue microenvironments, creating temporal and spatial discordance. To support explainability, data structures should encode temporal alignment, host‐microbe interaction matrices, and sampling metadata. Without such integration, XAI may conflate host susceptibility with microbial aggression under sparse data conditions. Structured data architectures enable attribution to defined holobiont interaction modules, supporting biologically interpretable mechanisms and precision management beyond opaque statistical correlations.

### Multi‐modal data fusion

Constructing a robust farm‐to‐fork XAI model necessitates the structured fusion of three primary data domains. First, host omics, including transcriptomics and metabolomics, capture ripening trajectories, membrane lipid remodeling, and defense metabolism, thereby encoding susceptibility to chilling injury and pathogen invasion. Second, microbiome profiles describe community composition and metabolic function, informing disease kinetics and host–pathogen competition. Third, the postharvest exposome encompasses continuous environmental sensor streams (temperature, humidity, O_2_/CO_2_, ethylene), event logs (harvest timing, precooling delay), and packaging barrier properties (oxygen transmission rate/water vapor transmission rate), which collectively determine modified‐atmosphere dynamics. Synthesizing these heterogeneous layers creates a complex multi‐modal landscape where standard modeling risks overfitting. Consequently, XAI is indispensable for validating these integrations. By enforcing feature attribution consistency, counterfactual reasoning, and physiological sanity checks, XAI verifies that multimodal models prioritize biological thresholds over spurious correlations. This transforms opaque predictions into trustworthy decision logic, ensuring interventions align with underlying physical reality rather than mere statistical artifacts.

### Data heterogeneity

Multi‐omics matrices and postharvest sensor streams differ substantially in sampling frequency, dimensionality, noise structure, and missingness, creating both computational and interpretability challenges. Variables such as cultivar, harvest maturity, orchard stress history, packaging batch, and cold‐chain interruptions may confound decay outcomes, causing XAI attributions to reinforce correlations rather than identify mechanistic drivers. Therefore, data engineering must be aligned with causal interpretability. Instead of merging high‐frequency sensor streams directly with omics matrices, sensor windows, omics anchor points, event logs, and packaging metadata should be organized into a causal‐ready schema that links exposure histories to each biological sampling point and preserves temporal ordering for downstream counterfactual reasoning. To address this complexity, we propose a data lakehouse architecture as the foundational infrastructure for postharvest XAI. By linking microbiome profiles, environmental sensor streams, packaging parameters, imaging data, and omics samples through shared batch identifiers and timestamped event anchors, the lakehouse enables sensor signals to be aggregated into biologically meaningful windows while preserving data provenance, missingness information, and temporal structure. Under this architecture, XAI can operate on harmonized, causally structured datasets, allowing attributions to be traced across biological pathways and logistical events and supporting mechanism‐oriented preservation decisions.

## XAI‐DRIVEN CAUSAL MECHANISM DISCOVERY IN POSTHARVEST SYSTEMS

### From correlation to causality in postharvest AI

In postharvest physiology, predictive accuracy alone rarely yields mechanistic insight because machine learning models often capture correlations within highly confounded biological systems. Predictive biomarkers may function only as proxies; for example, elevated lipoxygenase activity may correlate with soft rot, while the causal driver is prior temperature abuse. Similarly, citrus metabolomics combined with machine learning detected infection within 24 h after inoculation, but mechanistic interpretation required mapping predictive metabolites to phenylpropanoid and hormone–biosynthesis pathways. XAI helps address this limitation by identifying influential wavelengths, genes, metabolites, or microbial features and linking them to biologically coherent modules. When integrated with pathway analysis, experimental validation, and counterfactual reasoning, XAI can convert predictive features into testable hypotheses and bridge the gap between associative modeling and mechanism‐guided postharvest intervention.

### Translating XAI outputs into testable mechanisms

XAI enables predictive models to generate biologically interpretable hypotheses that can guide experimental validation in postharvest systems. For example, metabolomics integrated with machine learning has been used to detect latent infection in citrus fruit before visible decay [17]. Pathway enrichment analysis further linked the predictive metabolites to phenylpropanoid metabolism and hormone biosynthesis pathways, both central to plant defense responses. These findings illustrate how explainable models can project statistical features onto physiologically meaningful biochemical modules. Importantly, such attribution enables identification of early metabolic markers of pathogen invasion and provides targets for interventions, including elicitor treatments, antioxidant coatings, or modified‐atmosphere storage.

Evidence further demonstrates the mechanistic value of XAI in multi‐omics analysis. A comprehensive review summarized more than 40 deep‐learning studies applying attribution techniques such as saliency maps, feature importance scores, and attention mechanisms to identify regulatory DNA motifs and gene interactions underlying phenotype prediction [18]. In multi‐omics modeling, explainable pipelines have also identified compact, biologically meaningful feature sets from thousands of variables. For instance, graph‐based explainable models integrating transcriptomic and genomic datasets have successfully prioritized tens of candidate genes from datasets containing >10,000 features, linking model predictions to known regulatory pathways [19]. Together, these studies suggest a generalizable workflow for translating XAI outputs into mechanistic insight. Influential features identified by explainable models are first mapped onto biological pathways or microbial interaction modules. Candidate interventions such as adjusting storage atmosphere, applying antioxidants, or manipulating microbial communities are then defined. Finally, controlled experiments test whether perturbing the proposed mediator alters the predicted outcome. Through this feature‐to‐mechanism framework, XAI bridges statistical prediction and causal experimentation, enabling the development of actionable preservation strategies in postharvest biology [20].

## CHALLENGES AND FUTURE PERSPECTIVES

### Data standardization and interdisciplinary workflow

Despite rapid advances in multi‐omics modeling and digital twin simulations, cross‐laboratory and cross‐platform heterogeneity remains a major barrier to translation. Differences in sampling protocols, omics platforms, sensor calibration, and metadata standards complicate integration across supply‐chain stages. In multi‐omics contexts, high dimensionality combined with limited sample sizes further amplifies the risk of overfitting and confounding. Models with excessive parameterization may exhibit impressive internal performance while lacking stability across seasons, cultivars, or storage facilities. To address these challenges, integration strategies should prioritize module‐level fusion, which means aggregating features into biologically coherent pathway or microbial network modules rather than relying on unconstrained feature‐level models. Stability auditing, external validation, and cross‐domain transfer testing should be embedded by design. Without structured collaboration and standardization, AI models remain exploratory dashboards rather than decision‐grade tools.

### Edge deployment and explainability for instrumentation design

XAI offers a direct pathway from interpretation to deployment, particularly in spectroscopic quality assessment. Attribution analyses identifying informative wavelength bands have enabled rational sensor simplification, reducing hyperspectral inputs to compact band sets suitable for portable and low‐cost devices. In this context, explainability becomes an engineering constraint rather than a retrospective explanation. A similar logic applies to packaging sensors and cold‐chain IoT systems. XAI should specify which environmental channels and which exposure windows are mechanistically informative. In practice, however, explanation is more realistically performed in cloud or offline settings, whereas edge devices primarily execute lightweight inference using preselected features, compressed models, or explanation‐guided design rules. Edge‐deployable models must therefore integrate interpretability with computational efficiency, ensuring that actionable signals guide device architecture and logistics policies.

### Causal AI as the bridge to operable mechanism

The shift from prediction to intervention aligns with broader developments in AI‐for‐Science, where causal representation learning and counterfactual reasoning are increasingly emphasized. As model scale and multimodal integration expand, interpretability and causality become prerequisites for scientific credibility. For postharvest systems, this translates into an agenda of causal models that integrate physics‐informed constraints such as modified atmosphere packaging dynamics with holobiont‐aware omics signals. Counterfactual explanations must be embedded within structural causal models and validated experimentally to distinguish correlated predictors from manipulable mediators.

Postharvest preservation is entering a new phase in which AI must progress beyond prediction toward mechanistic understanding and actionable intervention. This perspective highlights XAI as a critical enabler of this transition. By integrating holobiont‐aware multi‐omics, multimodal sensing, and supply‐chain exposome data, XAI provides the analytical framework required to interpret complex biological and environmental interactions governing fruit deterioration. Ultimately, XAI offers a pathway toward mechanistically grounded, auditable, and deployable AI systems for postharvest management. Such advances are essential for transforming digital predictions into real‐world preservation strategies capable of reducing global food losses and improving supply‐chain sustainability.

## AI STATEMENT

AI tools were used exclusively for language editing and image refinement suggestion, with all scientific content and conclusions remaining the sole responsibility of the authors, and all AI‐related rights, disclosures, and compliance terms permitted under the publisher's policy are fully acknowledged and upheld.

## Supporting information


XAI Tutorial for absolute beginners.

## Data Availability

Data sharing is not applicable to this article as no datasets were generated or analyzed during the current study. No new data were generated in this review. Supplementary materials (XAI tutorial for absolute beginners, graphical abstract, slides, videos, Chinese translated version, and updated materials) may be found in the online DOI or iMeta Science http://www.imeta.science/.
