# Supplementary material for: From prediction to actionable mechanisms: Explainable multi‑omics AI for farm‑to‑fork postharvest preservation
Source: Imeta. 2026 Jun 9;5(3):e70139. doi: 10.1002/imt2.70139 (PMC13377414; doi:10.1002/imt2.70139)
Supplement: Supplementary file 1 — XAI Tutorial for absolute beginners. [file IMT2-5-e70139-s001.docx]

Supporting Information to

**From prediction to actionable mechanisms: Explainable multi‑omics AI for farm‑to‑fork postharvest preservation**

**Running title**: XAI-driven multi-omics for actionable postharvest preservation

Peihua Ma^1*^, Xiaoxue Jia^1^, Bei Fan^1^, Boqiang Li^2^, Tao Lin^3^, Jiping Sheng^4^, Cheng-I Wei^5^, Yingjian Lu^6^, Yizhou Ma^7^, Lin Chen^8^, Songtao Jiu^9^, Fengzhong Wang^1^^*^

^1^Institute of Food Science and Technology, Chinese Academy of Agricultural Sciences, Key Laboratory of Agro-products Processing, Ministry of Agriculture and Rural Affairs, Beijing 100193, China

^2^State Key Laboratory of Plant Diversity and Specialty Crops, Institute of Botany, Chinese Academy of Sciences, Beijing 100093, China

^3^Beijing Key Laboratory of Growth and Developmental Regulation for Protected Vegetable Crops, College of Horticulture, China Agricultural University, Beijing 100193, China

^4^School of Agricultural Economics and Rural Development, Renmin University of China, Beijing 100872, China

^5^Department of Nutrition and Food Science, College of Agriculture and Natural Resources, University of Maryland, College Park, College Park, MD 20742, USA

^6^College of Food Science and Engineering, Nanjing University of Finance and Economics, Nanjing 210003, China

^7^Laboratory of Food Process Engineering, Wageningen University & Research, P.O. Box 17, 6700 AA Wageningen, The Netherlands

^8^School of Chemistry, Chemical Engineering and Biotechnology, Nanyang Technological University, Singapore 637459, Singapore

^9^Department of Plant Science, School of Agriculture and Biology, Shanghai Jiao Tong University, Shanghai 200240, China

*Correspondence: mapeihua@caas.cn (Peihua Ma), wangfengzhong@sina.com (Fengzhong Wang)

**XAI TUTORIAL FOR ABSOLUTE BEGINNERS**

**Beginner level: What XAI is and what it is not**

Explainability refers to methods that provide human-understandable accounts of a model’s predictions (local explanations) or overall logic (global explanations). In biological systems, including postharvest physiology, the explanatory layer is often more valuable than the predictive output itself. For instance, a model predicting accelerated decay is useful, but understanding which metabolic pathway, microbial shift, or environmental stress signal drives that prediction is transformative [1].

At the foundational level, XAI should be regarded as a structured approach to probing feature influence within a trained model. Methods such as SHAP (SHapley Additive exPlanation) conceptually operate through feature removal or perturbation analyses, estimating the contribution of specific inputs, whether genes, metabolic pathways, or environmental variables, to model outputs. However, these attributions represent statistical importance rather than causal inference. For instance, if lipid oxidation markers are identified as strongly associated with predicted spoilage risk, this finding constitutes a correlation-based hypothesis that requires experimental validation through targeted perturbation, enzyme inhibition, or antioxidant intervention. Accordingly, early adoption of XAI in postharvest research must maintain epistemic rigor that explanations serve as hypothesis generators, not mechanistic proof [2].

**Intermediate level: Modality-specific XAI in postharvest research**

At the intermediate stage, researchers apply XAI toolkits aligned with specific data modalities common in postharvest systems.

**Tabular omics and engineered features.** Shapley-based approaches such as SHAP provide additive feature attributions that can identify differential genes, metabolites, or microbial taxa in multi-omics decay models. For example, SHAP ranking may reveal that genes associated with ethylene biosynthesis or specific fungal taxa drive predicted shelf-life reduction [3]. LIME (Local Interpretable Model-Agnostic Explanations) and perturbation-based surrogates approximate local model behavior but can become unstable in high-dimensional omics manifolds, especially when feature correlations reflect underlying metabolic coupling.

**Spectroscopy and imaging.** Agricultural spectroscopy represents one of the most practical domains of XAI deployment because explanations directly inform instrumentation design. Identifying informative wavelength bands allows simplification of hyperspectral systems into portable, low-cost devices for in-field quality detection. Gradient-based methods such as Integrated Gradients (IG) often provide more stable spectral attributions than raw saliency maps. Similarly, Gradient-weighted Class Activation Mapping (Grad-CAM) highlights spatial regions in fruit imaging, for example localized bruising or fungal colonization, linking CNN (convolutional neural network) predictions to visible structural degradation. In these contexts, XAI facilitated more efficient hardware optimization and contributed to cost reduction [4,5].

**Graph and network-based multi-omics integration.** Postharvest deterioration is inherently network-driven, involving host metabolic pathways, microbial community dynamics, and environmental stress signals. Graph neural networks (GNNs) increasingly model such interactions. Explainable graph methods, including GNNExplainer, identify influential subgraphs rather than isolated features, aligning naturally with pathway modules or microbial interaction networks. Multi-task architectures such as UnitedNet demonstrate multimodal fusion across transcriptomics and other modalities and allow post-hoc dissection of cross-modal relevance. In postharvest contexts, such approaches enable quantification of which host-microbe interaction modules drive decay under specific storage conditions [6].

**Professional stage: Counterfactual reasoning, auditing, and mechanism-oriented deployment in postharvest systems**

At the professional stage, XAI in postharvest research evolves from descriptive attribution toward intervention-oriented decision frameworks that integrate counterfactual reasoning, robustness auditing, and biological validation. Counterfactual explanation plays a central role by identifying feasible changes in input variables such as oxygen transmission rate (OTR), relative humidity, or specific metabolite concentrations that would alter predicted spoilage risk. In postharvest management environments, this capability enables scenario testing before physical intervention. However, counterfactual recommendations must adhere to physical and biological constraints (*e.g.*, material permeability, safety standards).

Professional deployment of XAI in postharvest multi-omics and multimodal modeling requires explanation auditing across four linked criteria: stability, faithfulness, interpretability, and biological plausibility. Gradient-based methods such as IG and guided backpropagation are computationally efficient and well suited to deep multimodal architectures, but their attributions can vary across random seeds, baseline choices, and correlated omics features, limiting stability in small-sample settings. Shapley-based approaches provide stronger local feature accounting and are often more intuitive for tabular omics integration, yet they are computationally expensive, sensitive to feature dependence, and can become unstable under severe high dimensionality and bootstrap resampling [7]. Perturbation-based methods better probe faithfulness because masking influential variables should alter spoilage predictions, but unrealistic perturbations may distort biological structure and exaggerate importance in tightly coupled molecular networks [8]. Attention scores and graph explainers can improve pathway-level interpretability for temporally or mechanistically connected data, although attention is not inherently faithful and graph explanations depend strongly on prior network quality [9]. These limitations are amplified in postharvest systems, where seasonal variation, cultivar effects, sparse sampling, and time-dependent ripening trajectories complicate attribution consistency. Accordingly, ensemble XAI frameworks such as OmiXAI are particularly valuable because they triangulate importance across complementary explainers, reduce method-specific bias, and compress thousands of candidate variables into biologically coherent targets that can be validated against KEGG (Kyoto Encyclopedia of Genes and Genomes), MetaCyc (Metabolic Pathways From all Domains of Life), and established ripening, oxidative, and defense pathways [9,10].

**Experimental validation, standardization, and governance**

To translate explainable AI outputs into actionable postharvest interventions, computational attribution must be coupled with standardized experimental validation across the supply chain. In packaging-centered preservation systems, candidate interventions, such as modified-atmosphere packaging (MAP) or humidity control, should be evaluated using quantifiable barrier parameters, including OTR and water vapor transmission rate, measured under recognized testing standards. Closing the mechanistic reasoning loop requires coordinated experimentation linking computational models with wet-lab and pilot-scale validation. A practical validation framework should proceed in four steps: (1) prioritize top XAI-derived features or pathways as testable hypotheses; (2) perturb them through controlled interventions, such as coating reformulation, microbial inoculation, or gene-targeted treatments; (3) quantify downstream effects on spoilage phenotypes, omics shifts, and model outputs using predefined effect-size and reproducibility criteria; and (4) verify generalizability across cultivars, storage regimes, and pilot-scale logistics. Counterfactual tests, temporal holdout validation, and orthogonal assays should be incorporated to distinguish causal drivers from correlative markers. Postharvest systems provide multiple intervention routes, including biochemical treatments (*e.g.*, antioxidant coatings or defense elicitors), microbial modulation through antagonistic consortia, and genetic strategies targeting ripening or senescence pathways. Iterative experimentation across storage, transport, and retail stages enables model refinement by updating causal pathways and improving prediction robustness under real supply-chain variability.

Governance and regulatory considerations must therefore be embedded throughout the XAI pipeline. Packaging innovations and storage interventions are subject to food-contact regulations across jurisdictions, while AI-assisted decision systems require transparent documentation of model validity, uncertainty, and operational boundaries. We propose assembling an integrated “evidence packet” for each actionable recommendation, including a model card describing intended use and uncertainty, a mechanism packet summarizing pathway-level explanations and stability audits, and a compliance packet containing packaging test reports and regulatory documentation. This governance framework should also clarify data ownership, data-sharing permissions, responsibility for model updates, and liability for intervention decisions among growers, logistics operators, retailers, and technology providers. Such integration ensures that explainable AI can support auditable, mechanistically grounded preservation strategies capable of reducing postharvest losses across the farm-to-fork supply chain.

**REFERENCE**

1. Patrizi, Gabriele, Luca Martiri, Antonio Pievatolo, Alessandro Magrini, Giovanni Meccariello, Loredana Cristaldi, Nedka D. Nikiforova. 2024. “A review of degradation models and remaining useful life prediction for testing design and predictive maintenance of lithium-ion batteries.” *Sensors* 24: 3382. https://doi.org/10.3390/s24113382

2. Afonso, Catarina B., Corinne M. Spickett. 2019. “Lipoproteins as targets and markers of lipoxidation.” *Redox Biology* 23: 101066. https://doi.org/10.1016/j.redox.2018.101066

3. Cheng, Yuan, Ying Wang, Lichuan Lou, Yuxuan Wang, Binger Dai, Yue Wang, Mariama Kebbeh, et al. 2024. “Transcriptomic analysis reveals the role of abscisic acid and ethylene in regulating starch-source biosynthesis associated with soft nose disorder in ‘Keitt’ mango fruit during postharvest.” *Postharvest Biology and Technology* 209: 112698. https://doi.org/10.1016/j.postharvbio.2023.112698

4. Hadiwijaya, Yuda, Ine Elisa Putri. 2025. “Spectroscopy in food and agriculture: A critical review of applications and adoption challenges.” *Food and Humanity* 5: 100800. https://doi.org/10.1016/j.foohum.2025.100800

5. Arrighi, Leonardo, Ingrid Alves de Moraes, Marco Zullich, Michele Simonato, Douglas Fernandes Barbin, Sylvio Barbon Junior. 2025. “Explainable Artificial Intelligence techniques for interpretation of food datasets: A review.” Preprint at *arXiv.* https://doi.org/10.48550/arXiv.2504.10527

6. Hernandez, Damian J., Aaron S. David, Eric S. Menges, Christopher A. Searcy, Michelle E. Afkhami. 2021. “Environmental stress destabilizes microbial networks.” *The ISME Journal* 15: 1722–1734. https://doi.org/10.1038/s41396-020-00882-x

7. Zou, Yuchen, Menglong Li, Tuerxunayi Abudumijiti, Huiming He, Mengying Guan, Yeerlin Asihaer, Miao Li, Nourhan M. Khattab, Mushui Shu, Yifei Hu. 2025. “The impact of school heavy metal exposure on children's gut microbiota: The mediating role of environmental microorganisms.” *iMeta* 4: e70021. https://doi.org/10.1002/imt2.70021

8. Peng, Xi, Kai Feng, Xingsheng Yang, Qing He, Bo Zhao, Tong Li, Shang Wang, Ye Deng. 2024. “iNAP 2.0: Harnessing metabolic complementarity in microbial network analysis.” *iMeta* 3: e235. https://doi.org/10.1002/imt2.235

9. Castellano-Escuder, Pol, Derek K. Zachman, Kevin Han, Matthey D. Hirschey. 2025. “GAUDI: interpretable multi-omics integration with UMAP embeddings and density-based clustering.” *Nature Communications* 16: 5771. https://doi.org/10.1038/s41467-025-60822-1

10. Huang, Jianyan, Xiaobo Zhao, Marco Bürger, Joanne Chory, Xinchao Wang. 2023. “The role of ethylene in plant temperature stress response.” *Trends in Plant Science* 28: 808–824. https://doi.org/10.1016/j.tplants.2023.03.001
